# Trends in initiation of regular cigarette smoking in 28 European countries, 1940–2019: retrospective reconstruction from repeated cross-sectional surveys

**DOI:** 10.1093/eurpub/ckaf184

**Published:** 2025-11-14

**Authors:** Ayaka Teshima, Olivia Bannon, Filippos T Filippidis, Ariadna Feliu, Silvano Gallus, Armando Peruga, Cristina Martínez, Esteve Fernández

**Affiliations:** Tobacco Control Unit, WHO Collaborating Centre for Tobacco Control, Catalan Institute of Oncology (ICO), L’Hospitalet de Llobregat, Barcelona, Spain; Tobacco Control Research Group, Institut d’Investigació Mèdica de Bellvitge—IDIBELL, L'Hospitalet de Llobregat, Barcelona, Spain; School of Medicine and Health Sciences, Campus de Bellvitge, Universitat de Barcelona, Barcelona, Spain; Tobacco Control Unit, WHO Collaborating Centre for Tobacco Control, Catalan Institute of Oncology (ICO), L’Hospitalet de Llobregat, Barcelona, Spain; Department of Primary Care and Public Health, School of Public Health, Imperial College London, London, United Kingdom; Department of Primary Care and Public Health, School of Public Health, Imperial College London, London, United Kingdom; Center for Biomedical Research in Respiratory Diseases (CIBER en Enfermedades Respiratorias, CIBERES), Madrid, Spain; Department of Medical Epidemiology, Istituto di Ricerche Farmacologiche Mario Negri IRCCS, Milan, Italy; Tobacco Control Unit, WHO Collaborating Centre for Tobacco Control, Catalan Institute of Oncology (ICO), L’Hospitalet de Llobregat, Barcelona, Spain; Tobacco Control Research Group, Institut d’Investigació Mèdica de Bellvitge—IDIBELL, L'Hospitalet de Llobregat, Barcelona, Spain; Center for Biomedical Research in Respiratory Diseases (CIBER en Enfermedades Respiratorias, CIBERES), Madrid, Spain; Centro de Epidemiología y Políticas de Salud, Universidad del Desarrollo Facultad de Medicina Clínica Alemana, Las Condes, Chile; Tobacco Control Unit, WHO Collaborating Centre for Tobacco Control, Catalan Institute of Oncology (ICO), L’Hospitalet de Llobregat, Barcelona, Spain; Tobacco Control Research Group, Institut d’Investigació Mèdica de Bellvitge—IDIBELL, L'Hospitalet de Llobregat, Barcelona, Spain; Center for Biomedical Research in Respiratory Diseases (CIBER en Enfermedades Respiratorias, CIBERES), Madrid, Spain; Department of Public Health, Mental Health and Maternal and Child Health Nursing, Faculty of Nursing, Universitat de Barcelona, Barcelona, Spain; Philip R. Lee Institute for Health Policy Studies, University of California San Francisco, San Francisco, CA, United States; Tobacco Control Unit, WHO Collaborating Centre for Tobacco Control, Catalan Institute of Oncology (ICO), L’Hospitalet de Llobregat, Barcelona, Spain; Tobacco Control Research Group, Institut d’Investigació Mèdica de Bellvitge—IDIBELL, L'Hospitalet de Llobregat, Barcelona, Spain; School of Medicine and Health Sciences, Campus de Bellvitge, Universitat de Barcelona, Barcelona, Spain; Center for Biomedical Research in Respiratory Diseases (CIBER en Enfermedades Respiratorias, CIBERES), Madrid, Spain; Secretariat of Public Health, Department of Health, Generalitat de Catalunya, Barcelona, Spain

## Abstract

In the European Union (EU), one in five youth currently smoke, with over half establishing regular smoking by age 18. Yet, evidence on the historical trends of smoking initiation remains scarce despite its importance for tobacco control. Using four waves of the Special Eurobarometer survey (2012–20; *n = *110 753, aged ≥15), we retrospectively estimated trends in initiation rates (IRs) of regular cigarette smoking in the EU from 1940 to 2019 among individuals aged 10–24 by sex, region, and country for each calendar decade. EU-wide smoking IRs have decreased compared to the peak period, with narrowing disparities by sex and region. For males, the IRs have declined from 5.7% (95% CI = 5.6–5.9) in the 1970s to 3.2% (95% CI = 3.0–3.3) in the 2010s, and for females from 3.9% (95% CI = 3.7–4.0) in the 1990s to 2.4% (95% CI = 2.3–2.5) in the 2010s. The decline was more pronounced among young adults aged 18–24 than minors aged 10–17, with minors’ IRs surpassing those of young adults during the 2010s. Marked declines occurred among young adults in all regions, while among minors, a clear decrease was observed only for males in Northern Europe. Concerningly, the IRs among minors have trended upward in Eastern Europe for both sexes. Despite declining youth smoking initiation, an unacceptably high number of European youth still begin smoking regularly before the legal age of 18. Stricter and comprehensive tobacco control policies targeting youth, along with smoke-free generation initiatives, could substantially reduce future tobacco use and smoking-related mortality.

## Introduction

Despite the decline in adult smoking prevalence in the European Union (EU), from 39.4% in 2002 to 24% in 2023, tobacco use among adolescents remains a major public health concern [1]. Existing literature on the EU has reported that, as of 2023, 40% of individuals who currently smoke began smoking regularly between ages 15 and 18, and 14% before age 15 [1], and has further estimated that over 172 000 15-year-old smokers have moderate to severe nicotine dependence [2].

The EU data have suggested that youth are initiating nicotine and tobacco products at a younger age and at higher rates than previous generations [3]. Transition from experimentation to regular smoking is a major concern, as it leads to increased risks of many smoking-related diseases [4]. Individuals who begin smoking before age 18 are more likely to become heavy smokers, smoke for a longer duration, and present lower successful quitting rates [5–7]. Age at smoking initiation is an important predictor of lifetime tobacco use and nicotine dependence and the associated health burden [6, 7].

While some existing tobacco control policies have discouraged adolescents from trying nicotine products, many are primarily effective in preventing those who experiment from becoming regular users [8]. In 2021, the EU Europe’s Beating Cancer Plan set a goal to achieve a “Tobacco-Free Generation,” aiming to reduce the prevalence of tobacco use to below 5% by 2040 [9]. “Stopping the start,” or, in other words, preventing adolescents from taking up smoking, is key to meeting this goal.

The EU has implemented various tobacco control measures through key legislation, such as the Tobacco Tax Directive (2011/64/EU) and the Tobacco Products Directive (2014/40/EU), as well as ratified the World Health Organization Framework Convention on Tobacco Control (WHO FCTC) by all member states (MS) and the EU as a supranational entity [10]. However, variations in tobacco control policy implementation and in the stages of the cigarette epidemic by sex and region are likely to contribute to heterogeneous smoking patterns and smoking-related mortality [11–15]. Smoking prevalence, and consequently the associated health burden, depends on both initiation and cessation of smoking [16]. Most existing evidence on initiation trends in Europe is country-specific, limiting cross-country comparisons due to varying observation periods and methods [17–19]. Existing EU-level research is limited to a cohort study covering 1970–2009 [20], which did not capture earlier decades or recent policy impacts, especially since 2010, when key EU-wide tobacco control policies were enacted [10], leaving evidence on long-term trends scarce. Monitoring changes in age at smoking initiation could provide a better understanding of the future burden of smoking for the EU population and aid in the development of evidence-based interventions to reduce smoking.

Hence, we aimed to explore the historical patterns in initiation of cigarette smoking among individuals aged 10–24 years across the EU, and examined differences by sex, region, and country.

## Methods

### Data source and study design

Data were drawn from four waves of the Special Eurobarometer surveys conducted by the European Commission, using repeated cross-sectional designs with representative samples of EU residents aged ≥15 years. Fieldwork was conducted in February–March 2012 (wave 77.1, *n = *26 751), November–December 2014 (wave 82.4, *n = *27 801), March 2017 (wave 87.1, *n = *27 901), and August–September 2020 (wave 93.2, *n = *28 300). Each wave employed a consistent random multistage sampling method, with sampling points selected with a probability proportional to population size and density. Post-stratification and national weighting accounted for age, sex, and area of residence. Each wave approached ∼1000 participants per MS, with around 500 participants in smaller countries such as Cyprus, Luxembourg, and Malta. In the 2012, 2014, and 2017 waves, data were collected through face-to-face interviews in the appropriate national language using computer-assisted personal interview (CAPI). In 2020, due to the COVID-19 pandemic, face-to-face CAPI remained the primary methods where feasible, while several countries conducted surveys online using computer-assisted web interview (CAWI) [21]. We merged data from all four waves, covering the current 27 EU MS (EU 27) and the UK (which was still an EU MS during the survey period) [21]; Croatia was not included in wave 77.1 (2012) as it had not yet joined. The final analytic sample comprised 110 753 respondents (Supplementary Table S1).

### Measures

#### Tobacco smoking

All participants in the 2012 wave were asked, “Regarding smoking cigarettes, cigars or a pipe, which of the following applies to you?” In the 2014, 2017, and 2020 waves, the participants were asked, “Regarding smoking cigarettes, cigars, cigarillos or a pipe, which of the following applies to you? In this question and the following questions in this section, smoking cigarettes does not include the use of electronic cigarettes.” Despite slight wording differences in the question, the response options were consistent across all waves: “You currently smoke” (current smoking); “You used to smoke but you have stopped” (former smoking); and “You have never smoked” (never smoking).

#### Age at initiation of cigarettes smoking

Age at initiation of regular cigarette smoking was assessed by asking respondents who reported current or former smoking: “How old were you when you started smoking on a regular basis, i.e. at least once a week?” Regular cigarette smoking was therefore defined as smoking at least once per week. Numerical answers were recorded. The question and response format remained consistent across all waves.

#### Sociodemographic characteristics and regional level

Sociodemographic data included information on sex (male, female). According to the United Nations geoscheme, the EU MS and the UK were classified into four subregions [22]: Northern Europe (Denmark, Estonia, Ireland, Latvia, Lithuania, Finland, Sweden, the UK), Western Europe (Austria, Belgium, France, Germany, Luxembourg, The Netherlands), Southern Europe (Cyprus, Greece, Italy, Malta, Portugal, Slovenia, Spain, Croatia), and Eastern Europe (Bulgaria, Czech Republic, Hungary, Poland, Romania, Slovakia).

#### Initiation rates of regular cigarette smoking

Initiation rates of regular cigarette smoking (hereafter referred to as “initiation rates of smoking”) were defined as the percentage of individuals at risk of smoking (i.e. those who had not yet started smoking at the time) who initiated cigarette smoking regularly at a specific age in a given year. Birth year was calculated by subtracting the self-reported age of the respondents from the survey year, and the calendar year of initiation was derived by adding their reported age at initiation to the birth year. For each age between 10 and 24 years and each calendar year, the numerator was the number of individuals who initiated smoking in a given age and calendar year. The denominator included all individuals who were never smokers up to that age and calendar year; individuals who had already initiated regular smoking before that calendar year were excluded from the risk population. Thus, participants contributed to the denominator for calendar years they remained never smokers, and to the numerator once in the calendar year they first initiated. For example, in the 2012 wave, a respondent born in 1954 who initiated smoking at age 14 contributed once to the numerator (age 14 in 1968) and five times to the denominator (ages 10–14 in 1964–68, as a never smoker until initiation). A respondent of the same birth year who never smoked contributed to the denominator for 15 calendar years (ages 10–24, 1964–78), but never to the numerator. The analysis period was restricted to 1940–2019, as calendar years before 1940 could not be reliably reconstructed due to the limited number of older respondents, and years after 2019 could not be included because the most recent survey wave in the analysis was conducted in 2020.

This retrospective reconstruction method has been applied in previous studies to estimate initiation and incidence rates from cross-sectional data in the absence of original longitudinal information [17, 19, 23–26].

### Statistical analyses

We first calculated initiation rates (IRs, %) for each calendar year between 1940 and 2019. To increase stability and reduce random variation due to small cell counts in single calendar years, these were then aggregated into eight decade-long calendar periods (1940–1949; 1950–1959; 1960–1969; 1970–1979; 1980–1989; 1990–1999; 2000–2009; 2010–2019). For each decade, we summed the numerators and denominators across years and calculated IRs with 95% confidence intervals (CIs), stratified by sex and subregion. To reduce age-heaping bias (tendency to report even-numbered ages than odd-numbered ages) [17–19, 24], we merged IRs into two-year intervals (10–11 and 23–24).

We additionally estimated IRs by age groups; overall aged 10–24, legal minors aged 10–17 and young adults aged 18–24 by sex and subregion in each decade. Country-specific IRs were calculated in four broader calendar periods (1940–59; 1960–79; 1980–99; 2000–19), instead of the eight periods due to a small national sample size.

The 95% CIs for IRs were calculated using the Wilson score method. Temporal changes in IRs were considered statistically significant when CIs did not overlap. All estimates applied official Eurobarometer weights to account for sampling design and ensure representativeness across EU MS. Analyses were conducted in R version 4.2.1.

A sensitivity analysis was conducted to assess whether estimation of IRs was influenced by the temporal distance between the survey year and the observation period. This analysis restricted the dataset to the earliest survey wave to assess potential biases in the reconstructed estimation of IRs for earlier decades.

## Results

The summary of sociodemographic characteristics of participants for the pooled sample and each wave is displayed in the Supplementary Table S2.

### General trends in smoking initiation among those aged 10–24

**Figure 1.**
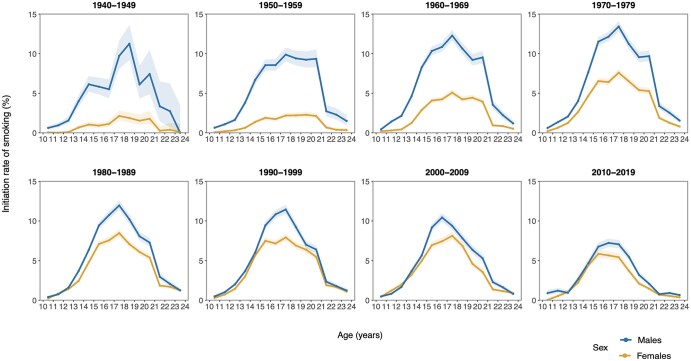
Initiation rates of smoking among those aged 10–24 by sex and calendar periods in the EU 27 MS and the UK from 1940 to 2019.

**Figure 2.**
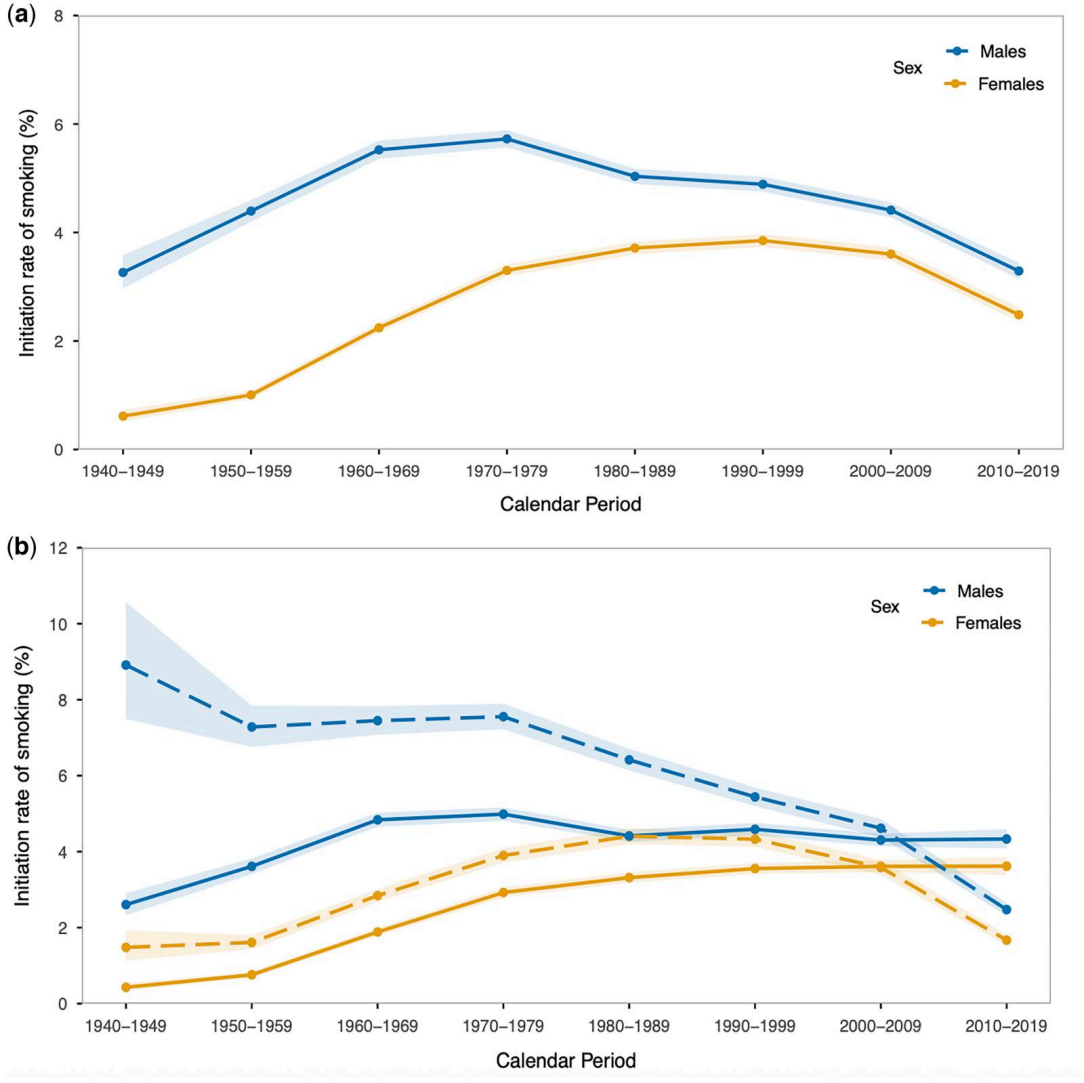
Initiation rates of smoking among those (a) aged 10–24, (b) aged 10–17 *versus* aged 18–24 by sex and calendar periods in the EU 27 MS and the UK from 1940 to 2019. Abbreviation: IRs = initiation rates of smoking. The solid line represents the group of legal minors aged 10–17, while the dashed line represents the group of young adults aged 18–24 for each decade.

Figure 1 shows the trends of IRs among 10- to 24-year-olds in the EU by sex from 1940 to 2019. The peak age at initiation remained stable around 17–18 years until the 1990s for males and the 2000s for females; thereafter, it shifted earlier to 16–17 years for males (IRs of 11.2% in the 2000s) and 15–16 years for females (IRs of 5.8% in the 2010s). IRs were consistently higher among males compared to females, with the sex difference most pronounced in the 1940s and narrowing over time due to larger declines in males. EU-overall IRs peaked at 5.7% (95% CI: 5.6–5.9) for males in the 1970s and 3.9% (95% CI: 3.7–4.0) for females in the 1990s, declining by the 2010s to 3.3% (95% CI: 3.1–3.4) and 2.5% (95% CI: 2.4–2.6), respectively (Fig. 2a and Supplementary Table S3).

### Trends in smoking initiation among minors versus young adults

Among young adults aged 18–24, IRs declined significantly from their peak levels in earlier decades: for males, from 7.6% (95% CI: 7.2–7.9) in the 1970s to 2.5% (95% CI: 2.3–2.6) in the 2010s, and for females, from 4.4% (95% CI: 4.2–4.6) in the 1980s to 1.7% (95% CI: 1.5–1.8) in the 2010s. In contrast, IRs among minors aged 10–17 rose from the 1960s and remained high thereafter. In the 2010s, for the first time, minors’ IRs exceeded those of young adults [males: 4.3% (95% CI: 4.1–4.6) *vs.* 2.5% (95% CI: 2.3–2.6); females: 3.6% (95% CI: 3.4–3.9) *vs.* 1.7% (95% CI: 1.5–1.8)] (Fig. 2b; Supplementary Table S3).

### Regional differences in smoking initiation

**Figure 3.**
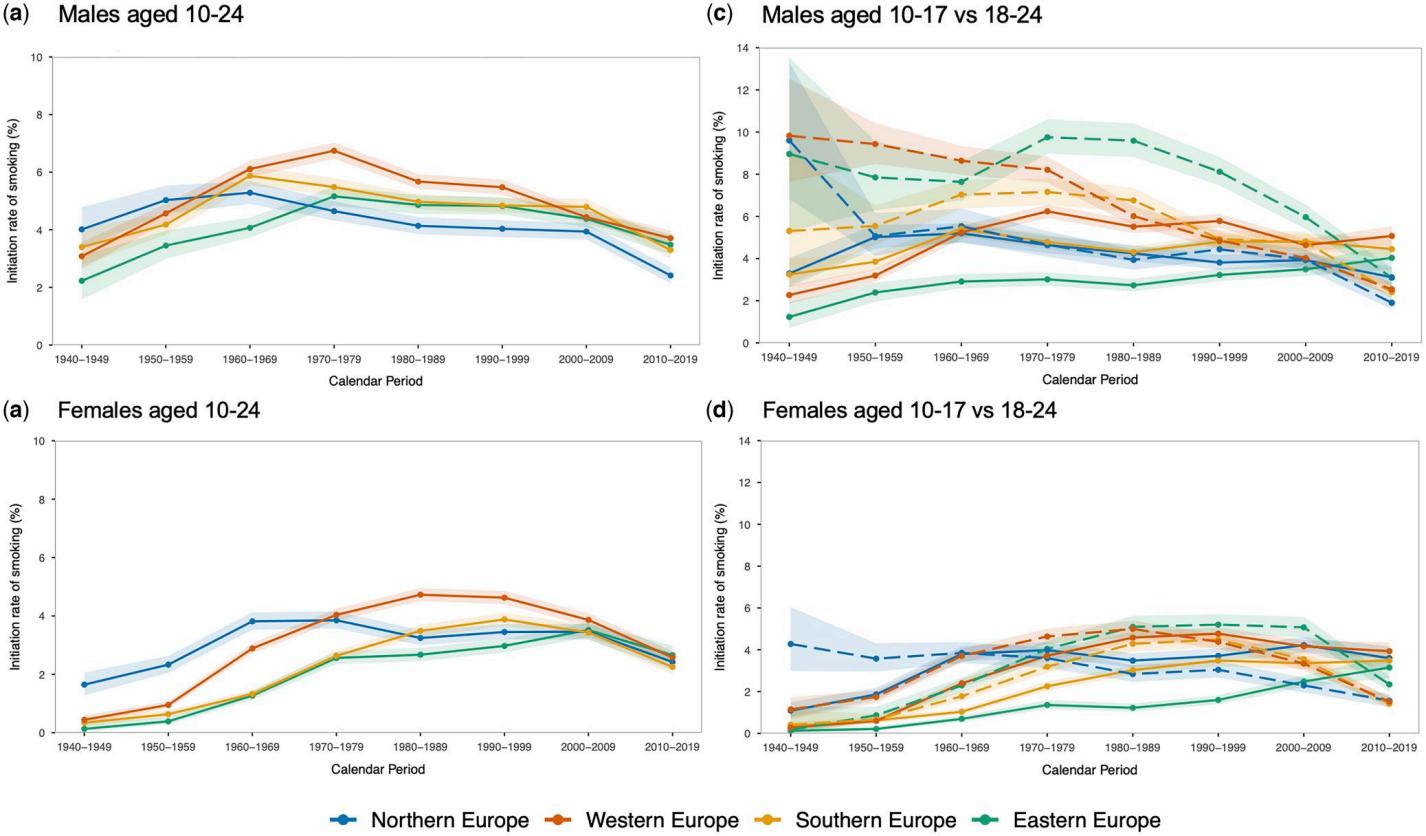
Initiation rates of smoking by age group, sex, and subregion in the EU (27 member states and the UK) from 1940 to 2019. Abbreviation: IR = initiation rates of smoking. The solid line represents the group of legal minors aged 10–17, while the dashed line represents the group of young adults aged 18–24 for each decade.

IRs among those aged 10–24 decreased across all regions, although peaks of initiation occurred in different periods: for males (Fig. 3a), in the 1960s in Northern (5.3%) and Southern Europe (5.9%) and the 1970s in Western (6.7%) and Eastern Europe (5.2%); for females (Fig. 3b), in the 1970s in Northern (3.9%), the 1980s in Western (4.7%), the 1990s in Southern (3.9%), and the 2000s in Eastern Europe (3.5%). In the 2010s, IRs among males aged 10–24 were significantly lower in Northern Europe (2.3%, 95% CI: 2.1–2.6) compared with Western (3.5%, 95% CI: 3.3–3.8), Southern (3.2%, 95% CI: 2.9–3.4), and Eastern Europe (3.4%, 95% CI: 3.1–3.8), whereas female IRs were similar across regions.

The overall decline was more rapid among young adults; this pattern observed across all regions and in both sexes (Fig. 3c and d). In the 2010s, minors’ IRs were significantly higher than those of young adults across all regions and sexes, except in Eastern Europe, where minors had a higher IR, but the 95% CIs overlapped, indicating no statistical significance.

Among male minors, IRs declined steadily in Northern Europe [from 5.2% (95% CI: 4.8–5.7) in the 1960s to 3.1% (95% CI: 2.6–3.6) in the 2010s] but showed a slight reduction in Western and Southern Europe and rose consistently in Eastern Europe. Among female minors, IRs slightly decreased but remained high in Northern and Western Europe, were stable in Southern Europe since the 1970s, and increased in Eastern Europe from the 1980s.

**Figure 4.**
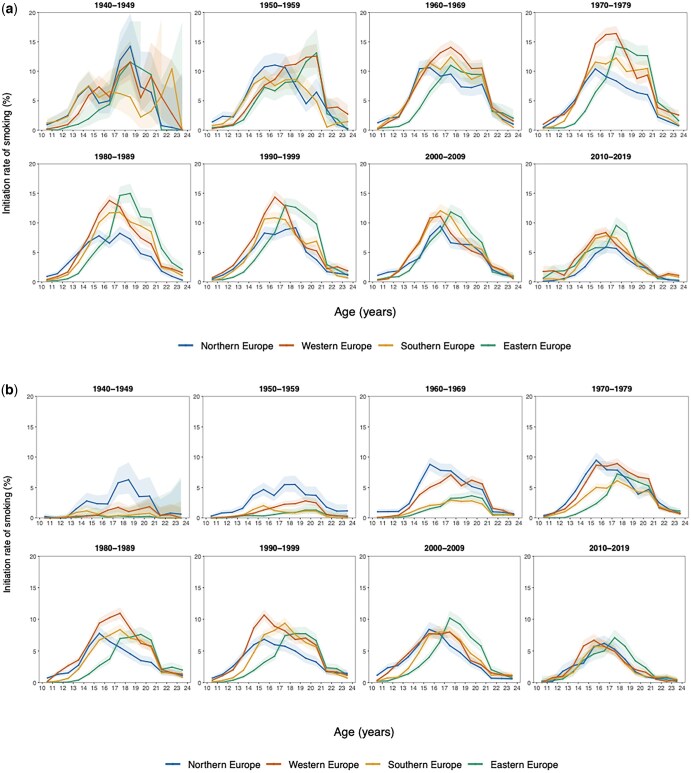
Initiation rates of smoking by sex and subregion in the EU (27 member states and the UK) from 1940 to 2019. (a) Males aged 10–24 and (b) females aged 10–24.

Initiation patterns varied markedly by region and sex. In Northern Europe, both males and females consistently initiated earlier, with peaks at ages 14–16, suggesting the earliest uptake compared to other regions. In Western Europe, the peak shifted from ages 17 to 18 in the 1960–70s to earlier ages in later decades, a trend similarly observed in Southern Europe, where peaks at 17–18 in the 1970s moved to 15–16 by the 2010s. In contrast, Eastern Europe consistently showed later initiation, with peaks around ages 17–19 for both sexes (Fig. 4a and b).

### Country differences in smoking initiation

A declining trend in IRs among males aged 10–24 was observed in most countries, with significant reductions over time in Denmark, Sweden, the UK, France, Germany, the Netherlands, and Spain (Supplementary Fig. S1a). Nevertheless, IRs among males aged 10–17 showed an upward trend in all Eastern European countries except for Poland as well as in some Northern European countries (e.g. Estonia, Latvia, and Lithuania). By contrast, IRs among females aged 10–24 have stagnated or increased in most countries except for Denmark, Sweden, the UK, France, Germany, and the Netherlands (Supplementary Fig. S1b). In particular, among females aged 10–17, significant declines were observed only in France and the Netherlands, whereas upward trends were evident in most other countries, especially in all Southern European countries except Spain and across all Eastern European countries. In the 2000s, Latvia and Bulgaria consistently showed the highest IRs across all age groups for both sexes.

## Discussion

### Main findings

This study revealed declining youth smoking initiation, with narrowing sex and regional disparities over the study period; however, there was a shift towards earlier ages of initiation. The decline was more pronounced among young adults (aged 18–24) compared to legal minors (aged 10–17), with minors’ rates surpassing those of young adults during the 2010s. The timing of peak initiation and pace of reduction also varied by regions and age groups. Among young adults marked declines were observed in all regions for both sexes. In contrast, among minors, clear decreases were found only for males in Northern Europe, while for females, initiation rates generally have stagnated or remained high across regions. Notably, initiation rates have increased in Eastern Europe for both sexes.

The observed overall decline aligns with international studies showing reduced youth smoking over time [3, 20, 27]. The European School Survey Project on Alcohol and Other Drugs (ESPAD) reported a 16% decrease in cigarette use among 15- to 16-year-olds between 1999 and 2019 [3]. This could be directly attributed to tobacco control efforts, such as all EU MS becoming Parties to the WHO FCTC since 2006, the introduction of MPOWER in 2007, and the tightening of EU-level tobacco control from 2010 onwards [10, 28, 29]. However, our findings show that reductions among young adults have primarily driven this decline, while initiation among minors has stagnated or even increased. This suggests that existing tobacco control measures have generally been effective for adults but may have had less impact on minors. Smoke-free environments and rising taxes may be effective in discouraging later uptake, particularly among “borderline” cases who experiment at a young age but progress to regular smoking later [8, 12]. In contrast, the most at-risk adolescents, those influenced by family or social environments, tend to initiate very early and are less responsive to such policies [12]. Moreover, recent evidence has suggested adolescents are highly susceptible to advertising, including social media and movies, highlighting the potential of marketing restrictions to reduce initiation [30].

Our results show that smoking initiation was predominantly concentrated among young adults for most of the study period but has gradually shifted toward minors. This shift became most evident in the 2010s, when minors’ rates exceeded those of young adults, reaching about twice as high in both sexes. This supports previous studies suggesting inadequate enforcement of age-of-sale laws [9, 31]. While most EU MS raised the legal age for tobacco sales from 16 to 18 years after 2010 [10, 31], the 2019 ESPAD survey found that about 60% of students reported easy access to tobacco [3], reflecting weaknesses in enforcement, such as poorly monitored stores or social sourcing [31]. Several US states have implemented “Tobacco 21” laws, raising the legal age to 21 which have proven effective in delaying initiation [31] and similar measures as part of smoke-free strategies could further limit adolescent access. Previous research has also suggested that the tobacco industry has historically sought to appeal to younger populations through marketing and product strategies [32, 33], which may contribute to persistence of initiation at minors, although this was not directly examined in our study.

Patterns in initiation showed peaks at different periods across regions (in order: Northern, Western, Southern, and Eastern Europe), with males experiencing the peak earlier than females. These patterns broadly mirror the stages of the cigarette epidemic and differences in tobacco control policy implementation between regions [14, 15]. While significant declines in initiation were seen among young adults across all regions, a clear decline among minors was found only for Northern European males. Elsewhere, minors’ initiation stagnated, and it increased for both sexes in Eastern Europe. These patterns are consistent with evidence showing rising early-adolescent initiation since the 1990s, except among Northern European males, suggesting persistent or worsening uptake in adolescence. These divergent trends may partly reflect historical differences in the smoking epidemic and policy response. In Northern Europe, where tobacco control measures have traditionally been strong [34], males were at the forefront of the epidemic [15] and therefore benefited earlier from taxation, advertising bans, and smoke-free policies. By contrast, delayed introduction and enforcement of tobacco control in Eastern Europe may explain the increasing initiation among minors in this region [14, 34].

Price differences may also play an important role: in the UK and Ireland, high taxation has kept cigarette prices elevated, whereas in Spain, Italy, and many Eastern European countries, prices have remained relatively low [35–37]. Since youth are particularly price-sensitive, these policy differences likely influenced both the timing and magnitude of smoking initiation [8, 38]. Furthermore, regional differences may also reflect underlying sociodemographic conditions, particularly the national economic context and educational attainment. High-Gross Domestic Product (GDP) countries, such as the UK and Ireland, have experienced earlier declines in initiation, while lower-GDP countries, such as Latvia and Lithuania, have shown slower progress despite being in the same region. Economic context may influence the affordability of tobacco, as well as the capacity to implement and enforce strong tobacco control measures [34, 37]. At the individual level, the smoking epidemic theory highlights sex and socioeconomic status (SES) as key determinants of smoking behaviour [15], and previous research has consistently shown that lower education is associated with earlier initiation and greater susceptibility to tobacco use [39, 40]. Although Eurobarometer provides data on education and financial difficulties, these variables capture SES at the time of survey rather than at initiation, limiting their value for retrospective patterns. More appropriate indicators, such as parental education, were unavailable. Future research should examine how socioeconomic disadvantage influences initiation over time.

To further curb smoking initiation in the EU, greater emphasis must be placed on preventing uptake among minors, who are especially susceptible to parental, peer, and social influences [12]. This highlights the need for targeted school-based and community interventions, alongside strengthened enforcement of age-of-sale laws, comprehensive bans on advertising and promotion (including on social media), and expansion of smoke-free environments [8, 11]. At the EU policy level, revising the Tobacco Tax Directive and the Tobacco Products Directive [9, 13] remains a priority, with particular attention to raising minimum excise taxes and harmonizing prices across all nicotine and tobacco products [8]. Regional disparities underscore the importance of tailoring implementation and monitoring, with stronger measures urgently needed in regions where progress has lagged. Ensuring equitable implementation across countries and population groups is necessary to prevent a new generation from taking up smoking and to achieve the EU goal of a smoke-free generation by 2040.

### Strengths and limitations

The study has several strengths. Using data from a consistent survey methodology and representative EU samples, we were able to generalize population trends in smoking initiation across MS. Our reconstruction approach allowed for some countries where past data were limited, facilitating the estimation of historical trends in youth smoking behaviour. This method has been validated in previous studies, yielding estimates consistent with our study [17, 19, 23–26]. Our findings should be interpreted with caution due to several limitations. Some recall bias cannot be disregarded, particularly among older respondents. However, previous research has shown that while retrospective reporting of initiation can introduce some bias, this mainly manifests as a preference for reporting rounded ages (e.g. 16, 18, 20 instead of 17 or 19) rather than systematic misreporting by age at interview [25]. To assess this, we conducted a sensitivity analysis restricting the dataset to the earliest survey wave, which yielded similar patterns across decades. Thus the overall potential influence of recall bias on our estimates is considered limited, consistent with previous findings [20]. Our study may have also underestimated the reconstructed IRs in earlier calendar periods (e.g. the 1940s and 1950s) compared to the IRs in recent periods due to survivorship bias. Differences in cohort age composition across calendar periods may also have affected IR estimation, with more recent periods consisting mainly of younger cohorts, whereas estimates of IRs for earlier calendar periods consisted of older cohorts. However, a previous study on the validity of the reconstruction method showed that differential mortality among smokers minimally affected the reconstructed rates [23]. Moreover, limited product-specific questions hindered analysis of newer products, such as heated tobacco products. While this might have influenced IR’s most recent periods, their overall impact is likely minor given the dominance of conventional cigarettes in the EU market [1, 10]. Finally, our results might not fully represent those countries with smaller sample sizes, like Cyprus, Malta, Croatia, and Luxembourg.

## Conclusions

Our study provides the most detailed evidence to date on patterns and long-term trends in smoking initiation in the EU over the past 80 years. While overall initiation rates have declined and disparities by sex and region have narrowed, an unacceptably high number of youths still begin smoking regularly before the legal age of 18, with concerning stagnation or increases among minors in initiation in some regions. These findings underscore the urgent need for stronger, tailored tobacco control measures to prevent youth smoking uptake and achieve the EU goal of a smoke-free generation by 2040.

## Supplementary Material

ckaf184_Supplementary_Data

## Data Availability

The datasets for this study are publicly available at the GESIS Data Archive. (https://www.gesis.org/en/home).
