# Supplementary material for: Trends in initiation of regular cigarette smoking in 28 European countries, 1940–2019: retrospective reconstruction from repeated cross-sectional surveys
Source: Eur J Public Health. 2025 Nov 14;35(6):1204–11. doi: 10.1093/eurpub/ckaf184 (PMC12707475; doi:10.1093/eurpub/ckaf184)
Supplement: ckaf184_Supplementary_Data [file ckaf184_supplementary_data.docx]

**Supplementary Files**

**Supplementary Table S1:** Final analysis sample size per country per survey year

**Supplementary Table S2:** Characteristics of the respondents of the Special Eurobarometer surveys on tobacco in 2012, 2014, 2017, and 2020

**Supplementary Table S3**: Initiation rates of regular smoking by age groups and sex in the EU (27 member states and the UK) from 1940 to 2019

**Supplementary Table S4:** Initiation rates of smoking by age group, sex and subregion in the EU (27 member states and the UK) from 1940 to 2019

**Supplementary Fig. S1a:** Heatmap of initiation rates of smoking by three age-groups, sex and country in 27 EU member states and the UK from 1940 to 2019 among males

**Supplementary Fig. S1b:** Heatmap of initiation rates of smoking by three age-groups, sex and country in 27 EU member states and the UK from 1940 to 2019 among females

**Supplementary Table S1. Final analysis sample size per country per survey year**

|  | 2012 | 2014 | 2017 | 2020 | **Total** |
| --- | --- | --- | --- | --- | --- |
| Austria | 1031 | 1044 | 1001 | 1008 | **4084** |
| Belgium | 1051 | 1009 | 1023 | 981 | **4064** |
| Bulgaria | 1006 | 1003 | 1044 | 1057 | **4110** |
| Croatia | - | 1009 | 1048 | 1019 | **3076** |
| Cyprus (Republic) | 506 | 500 | 501 | 505 | **2012** |
| Czech Republic | 1003 | 1044 | 1058 | 1027 | **4132** |
| Denmark | 1019 | 1024 | 1000 | 1022 | **4065** |
| Estonia | 1000 | 998 | 1017 | 1104 | **4119** |
| Finland | 1003 | 1010 | 1012 | 1099 | **4124** |
| France | 1059 | 1009 | 1004 | 1001 | **4073** |
| Germany | 1552 | 1572 | 1537 | 1527 | **6188** |
| Greece | 999 | 1008 | 1010 | 1016 | **4033** |
| Hungary | 1021 | 1057 | 1053 | 1058 | **4189** |
| Ireland | 1008 | 1003 | 1021 | 1239 | **4271** |
| Italy | 1036 | 1010 | 1022 | 1021 | **4089** |
| Latvia | 1024 | 1003 | 1004 | 1024 | **4055** |
| Lithuania | 1021 | 1007 | 1001 | 1008 | **4037** |
| Luxembourg | 501 | 504 | 510 | 608 | **2123** |
| Malta | 500 | 502 | 500 | 502 | **2004** |
| Poland | 1000 | 1012 | 1008 | 1057 | **4077** |
| Portugal | 1009 | 1002 | 1061 | 1061 | **4133** |
| Romania | 1020 | 1034 | 1033 | 1103 | **4190** |
| Slovakia | 1000 | 1031 | 1014 | 1046 | **4091** |
| Slovenia | 1017 | 1035 | 1027 | 1011 | **4090** |
| Spain | 1004 | 1011 | 1024 | 1049 | **4088** |
| Sweden | 1016 | 1029 | 1007 | 998 | **4050** |
| The Netherlands | 1014 | 1019 | 1015 | 1086 | **4134** |
| United Kingdom | 1331 | 1312 | 1346 | 1063 | **5052** |
| **Total** | **26751** | **27801** | **27901** | **28300** | **110,753** |

**Supplementary Table S2. Characteristics of the respondents of the Special Eurobarometer surveys on tobacco in 2012, 2014, 2017, and 2020**

|  | 2012 | 2014 | 2017 | 2020 |
| --- | --- | --- | --- | --- |
|  | N (weighted %) | N (weighted %) | N (weighted %) | N (weighted %) |
| **Sex** |  |  |  |  |
| Female | 14324 (51.8) | 15190 (51.8) | 15015 (51.7) | 14969 (51.4) |
| Male | 12093 (48.2) | 12370 (48.2) | 12339 (48.3) | 12744 (48.6) |
|  |  |  |  |  |
| **Age** |  |  |  |  |
| 15-24 | 2953 (14.4) | 2731 (13.6) | 2422 (13.0) | 2529 (12.7) |
| 25-34 | 3809 (15.4) | 3585 (14.7) | 3566 (14.8) | 3620 (14.4) |
| 35-44 | 4442 (17.3) | 4573 (18.1) | 4155 (16.4) | 4243 (16.2) |
| 45-54 | 4737 (17.9) | 4626 (17.1) | 4469 (17.2) | 4939 (17.7) |
| 55-64 | 4521 (14.4) | 4847 (14.9) | 5087 (14.7) | 5246 (15.8) |
| 65+ | 5955 (20.6) | 7198 (21.6) | 7655 (23.8) | 7134 (23.2) |
|  |  |  |  |  |
| **Education** |  |  |  |  |
| Lower | 4937 (21.1) | 4591 (18.4) | 4215 (16.9) | 3182 (12.8) |
| Upper | 12514 (47.0) | 12586 (46.7) | 12435 (45.6) | 12145 (45.5) |
| Higher | 8668 (30.8) | 9970 (33.3) | 10313 (35.8) | 11817 (39.8) |
| Other | 298 (1.1) | 413 (1.7) | 391 (1.6) | 571 (2.0) |
|  |  |  |  |  |
| **Area of residence** |  |  |  |  |
| Rural | 9431 (34.0) | 8328 (29.0) | 8903 (30.2) | 9256 (30.0) |
| Small-middle town | 9515 (40.0) | 11663 (45.5) | 10853 (44.7) | 9997 (42.4) |
| Urban | 7443 (25.9) | 7549 (25.4) | 7584 (25.1) | 8451 (27.7) |
|  |  |  |  |  |
| **Financial difficulties** |  |  |  |  |
| Almost never/never | 15605 (62.1) | 17419 (64.9) | 17539 (64.9) | 19260 (70.1) |
| From time to time/  most of the time | 10315 (35.2) | 9754 (33.1) | 9388 (32.6) | 8259 (29.0) |
|  |  |  |  |  |
| **Smoking status** |  |  |  |  |
| Never smokers | 13550 (51.2) | 15125 (54.7) | 14485 (53.3) | 15089 (55.5) |
| Current smokers | 7206 (27.7) | 6743 (26.0) | 6754 (26.1) | 6366 (22.7) |
| Former smokers | 5594 (20.9) | 5624 (19.1) | 6067 (20.3) | 6239 (21.7) |
|  |  |  |  |  |
| **Age at initiation** (mean) | 18.0 | 18.5 | 18.3 | 18.3 |
| **Age at initiation** (median) | 17.0 | 18.0 | 17.0 | 17.0 |

**Supplementary Table S3. Initiation rates of regular smoking by age groups and sex in the EU (27 member states and the UK) from 1940 to 2019**

| **Males** | **Aged 10-24** | **Aged 10-17** | **Aged 18-24** | **Females** | **Aged 10-24** | **Aged 10-17** | **Aged 18-24** |
| --- | --- | --- | --- | --- | --- | --- | --- |
| Calendar Period | Weighted % (95% CI) | | | Calendar Period | Weighted % (95% CI) | | |
| 1940-1949 | 3.3 (3.0-3.6) | 2.6 (2.3-2.9) | 8.9 (7.5-10.6) | 1940-1949 | 0.6 (0.5-0.7) | 0.4 (0.3-0.5) | 1.5 (1.1-1.9) |
| 1950-1959 | 4.4 (4.2-4.6) | 3.6 (3.4-3.8) | 7.3 (6.8-7.8) | 1950-1959 | 1.0 (0.9-1.1) | 0.8 (0.7-0.8) | 1.6 (1.4-1.8) |
| 1960-1969 | 5.5 (5.4-5.7) | 4.8 (4.7-5.0) | 7.4 (7.1-7.8) | 1960-1969 | 2.2 (2.2-2.3) | 1.9 (1.8-2.0) | 2.8 (2.7-3.0) |
| 1970-1979 | 5.7 (5.6-5.9) | 5.0 (4.8-5.2) | 7.6 (7.2-7.9) | 1970-1979 | 3.3 (3.2-3.4) | 2.9 (2.8-3.1) | 3.9 (3.7-4.1) |
| 1980-1989 | 5.0 (4.9-5.2) | 4.4 (4.3-4.6) | 6.4 (6.1-6.7) | 1980-1989 | 3.7 (3.6-3.8) | 3.3 (3.2-3.5) | 4.4 (4.2-4.6) |
| 1990-1999 | 4.9 (4.8-5.0) | 4.6 (4.4-4.8) | 5.4 (5.2-5.7) | 1990-1999 | 3.9 (3.7-4.0) | 3.6 (3.4-3.7) | 4.3 (4.1-4.5) |
| 2000-2009 | 4.4 (4.3-4.5) | 4.3 (4.1-4.5) | 4.6 (4.4-4.9) | 2000-2009 | 3.6 (3.5-3.7) | 3.6 (3.5-3.8) | 3.6 (3.4-3.8) |
| 2010-2019 | 3.3 (3.1-3.4) | 4.3 (4.1-4.6) | 2.5 (2.3-2.6) | 2010-2019 | 2.5 (2.4-2.6) | 3.6 (3.4-3.9) | 1.7 (1.5-1.8) |

**Supplementary Table S4. Initiation rates of smoking by age group, sex and subregion in the EU (27 member states and the UK) from 1940 to 2019**

|  | **Males** | | | | | | | | | | | | | | | | | | | | | | | | | |
| --- | --- | --- | --- | --- | --- | --- | --- | --- | --- | --- | --- | --- | --- | --- | --- | --- | --- | --- | --- | --- | --- | --- | --- | --- | --- | --- |
|  | **Northern Europe** | | | | | | **Western Europe** | | | | | | | | **Southern Europe** | | | | | **Eastern Europe** | | | | | | |
| **Calendar period** | **_10-24_ IRs**  **(95% CI)** | | **_10-17_ IRs** | **_18-24_ IRs** | | **_10-24_ IRs** | | | | **_10-17_ IRs** | **_18-24_ IRs** | | **_10-24_ IRs** | | | | **_10-17_ IRs** | **_18-24_ IRs** | | | **_10-24_ IRs** | | | **_10-17_ IRs** | | **_18-24_ IRs** |
| 1940-1949 | 4.0 (3.4-4.8) | | 3.3 (2.6-4.0) | 9.6  (6.8-13.3) | | 3.1 (2.7-3.6) | | | | 2.3 (1.9-2.7) | 9.8  (7.6-12.5) | | 3.4 (2.8-4.1) | | | | 3.2 (2.6-4.0) | 5.3 (2.8-9.1) | | | 2.2 (1.6-3.1) | | | 1.2 (0.7-1.9) | | 9.0 (5.3-13.5) |
| 1950-1959 | 5.0 (4.6-5.5) | | 5.0 (4.5-5.6) | 5.1 (4.1-6.2) | | 4.6 (4.3-4.9) | | | | 3.2 (2.9-3.5) | 9.4  (8.5-10.4) | | 4.2 (3.8-4.5) | | | | 3.9 (3.5-4.2) | 5.5 (4.7-6.5) | | | 3.5 (3.0-4.0) | | | 2.4 (2.0-2.9) | | 7.8 (6.3-9.6) |
| 1960-1969 | 5.3 (4.9-5.7) | | 5.2 (4.8-5.7) | 5.5 (4.8-6.4) | | 6.1 (5.8-6.4) | | | | 5.2 (4.9-5.6) | 8.6 (8.0-9.3) | | 5.9 (5.5-6.2) | | | | 5.4 (5.0-5.8) | 7.0 (6.4-7.7) | | | 4.1 (3.7-4.4) | | | 2.9 (2.6-3.3) | | 7.6 (6.8-8.6) |
| 1970-1979 | 4.6 (4.3-5.0) | | 4.6 (4.3-5.1) | 4.7 (4.1-5.3) | | 6.7 (6.5-7.0) | | | | 6.2 (5.9-6.6) | 8.2 (7.6-8.8) | | 5.5 (5.2-5.8) | | | | 4.8 (4.5-5.1) | 7.2 (6.5-7.8) | | | 5.2 (4.8-5.5) | | | 3.0 (2.7-3.4) | | 9.8 (9.0-10.6) |
| 1980-1989 | 4.1 (3.9-4.4) | | 4.2 (3.9-4.6) | 3.9 (3.5-4.4) | | 5.7 (5.4-5.9) | | | | 5.5 (5.2-5.8) | 6.0 (5.6-6.5) | | 5.0 (4.7-5.2) | | | | 4.3 (4.0-4.6) | 6.8 (6.2-7.3) | | | 4.9 (4.6-5.2) | | | 2.7 (2.4-3.0) | | 9.6 (8.8-10.4) |
| 1990-1999 | 4.0 (3.7-4.3) | | 3.8 (3.5-4.2) | 4.4 (3.9-5.0) | | 5.5 (5.2-5.7) | | | | 5.8 (5.5-6.1) | 4.9 (4.4-5.3) | | 4.8 (4.6-5.1) | | | | 4.8 (4.5-5.1) | 4.9 (4.5-5.3) | | | 4.8 (4.5-5.1) | | | 3.2 (2.9-3.5) | | 8.1 (7.5-8.8) |
| 2000-2009 | 3.9 (3.7-4.2) | | 3.9 (3.6-4.3) | 4.0 (3.5-4.4) | | 4.4 (4.2-4.7) | | | | 4.6 (4.4-4.9) | 4.0 (3.7-4.4) | | 4.8 (4.5-5.1) | | | | 4.8 (4.5-5.2) | 4.8 (4.3-5.3) | | | 4.4 (4.1-4.7) | | | 3.5 (3.2-3.8) | | 6.0 (5.4-6.5) |
| 2010-2019 | 2.3 (2.1-2.6) | | 3.1 (2.7-3.6) | 1.9 (1.6-2.2) | | 3.5 (3.3-3.8) | | | | 5.1 (4.6-5.5) | 2.5 (2.3-2.8) | | 3.2 (2.9-3.4) | | | | 4.5 (4.0-5.0) | 2.4 (2.1-2.8) | | | 3.4 (3.1-3.8) | | | 4.0 (3.5-4.7) | | 3.1 (2.7-3.5) |
|  | **Females** | | | | | | | | | | | | | | | | | | | | | | | | | |
|  | **Northern Europe** | | | | | | | **Western Europe** | | | | | | **Southern Europe** | | | | | | | | **Eastern Europe** | | | | |
| **Calendar period** | **_10-24_ IRs** | **_10-17_ IRs** | | | **_18-24_ IRs** | | | **_10-24_ IRs** | **_10-17_ IRs** | | | **_18-24_ IRs** | | **_10-24_ IRs** | | **_10-17_ IRs** | | | **_18-24_ IRs** | | | **_10-24_ IRs** | **_10-17_ IRs** | | **_18-24_ IRs** | |
| 1940-1949 | 1.7 (1.3-2.1) | | 1.1 (0.8-1.5) | 4.3 (3.0-6.0) | | 0.4 (0.3-0.6) | | | | 0.3 (0.2-0.4) | 1.1 (0.7-1.7) | | 0.3 (0.2-0.5) | | | | 0.3 (0.2-0.5) | 0.4 (0.1-1.1) | | | 0.1 (0.0-0.4) | | | 0.1 (0.0-0.4) | | 0.2 (0.0-1.3) |
| 1950-1959 | 2.3 (2.1-2.6) | | 1.9 (1.6-2.2) | 3.6 (3.0-4.3) | | 1.0 (0.8-1.1) | | | | 0.6 (0.5-0.7) | 1.7 (1.5-2.1) | | 0.6 (0.5-0.8) | | | | 0.6 (0.5-0.8) | 0.7 (0.5-0.9) | | | 0.4 (0.3-0.5) | | | 0.2 (0.1-0.3) | | 0.9 (0.6-1.3) |
| 1960-1969 | 3.8 (3.5-4.1) | | 3.8 (3.4-4.2) | 3.8 (3.4-4.4) | | 2.9 (2.7-3.1) | | | | 2.4 (2.2-2.6) | 3.7 (3.4-4.0) | | 1.3 (1.2-1.5) | | | | 1.0 (0.9-1.2) | 1.8 (1.6-2.0) | | | 1.3 (1.1-1.4) | | | 0.7 (0.6-0.9) | | 2.3 (2.0-2.7) |
| 1970-1979 | 3.9 (3.6-4.2) | | 4.0 (3.6-4.4) | 3.6 (3.1-4.1) | | 4.0 (3.8-4.2) | | | | 3.7 (3.5-4.0) | 4.6 (4.3-5.0) | | 2.6 (2.5-2.8) | | | | 2.3 (2.1-2.5) | 3.2 (2.9-3.5) | | | 2.6 (2.4-2.8) | | | 1.4 (1.2-1.6) | | 4.0 (3.7-4.5) |
| 1980-1989 | 3.2 (3.0-3.5) | | 3.5 (3.2-3.8) | 2.8 (2.5-3.3) | | 4.7 (4.5-5.0) | | | | 4.6 (4.3-4.9) | 5.0 (4.6-5.4) | | 3.5 (3.3-3.7) | | | | 3.0 (2.8-3.3) | 4.3 (3.9-4.7) | | | 2.7 (2.5-2.9) | | | 1.2 (1.0-1.4) | | 5.1 (4.6-5.6) |
| 1990-1999 | 3.5 (3.2-3.7) | | 3.7 (3.4-4.1) | 3.0 (2.7-3.5) | | 4.6 (4.4-4.9) | | | | 4.8 (4.5-5.1) | 4.4 (4.0-4.8) | | 3.9 (3.7-4.1) | | | | 3.5 (3.2-3.8) | 4.5 (4.1-4.9) | | | 3.0 (2.8-3.2) | | | 1.6 (1.4-1.8) | | 5.2 (4.7-5.7) |
| 2000-2009 | 3.5 (3.2-3.7) | | 4.2 (3.9-4.6) | 2.3 (2.0-2.7) | | 3.9 (3.7-4.1) | | | | 4.2 (3.9-4.4) | 3.3 (3.0-3.7) | | 3.4 (3.2-3.7) | | | | 3.4 (3.1-3.7) | 3.5 (3.2-3.9) | | | 3.5 (3.3-3.8) | | | 2.5 (2.2-2.8) | | 5.1 (4.6-5.6) |
| 2010-2019 | 2.3 (2.1-2.6) | | 3.6 (3.1-4.2) | 1.6 (1.3-1.9) | | 2.5 (2.3-2.7) | | | | 3.9 (3.5-4.4) | 1.5 (1.3-1.8) | | 2.2 (1.9-2.4) | | | | 3.5 (3.0-4.0) | 1.4 (1.2-1.7) | | | 2.6 (2.3-2.9) | | | 3.2 (2.7-3.7) | | 2.3 (2.0-2.7) |

Footnote: IRs=Initiation rates of smoking. 95%CI=95% confidence intervals. _10-24_ IRs=Initiation rates of smoking among those who aged 10-24. _10-17_ IRs= Initiation rates of smoking among those who aged 10-17. _18-24_ IRs=Initiation rates of smoking among those who aged 18-24.

**Supplementary Fig. S1. Heatmap of initiation rates of smoking by three age-groups, sex and country in 27 EU member states and the UK from 1940 to 2019**

**Supplementary Fig. S1a. Males**

**Supplementary Fig. S1b. Females**
